# Hydroxychloroquine is safe and efficacious in oral lichen planus: data from a large outpatient cohort

**DOI:** 10.1007/s00403-025-04226-7

**Published:** 2025-04-15

**Authors:** Khava Abdusalamova, Margitta Worm, Farzan Solimani

**Affiliations:** Division of Allergy and Immunology, Department of Dermatology, Venereology and Allergology, Charité - Universitätsmedizin Berlin, Charitéplatz 1, 10117 Berlin, Germany

**Keywords:** Lichen planus, Therapy, Hydroxychloroquine, Retrospective study

## Abstract

Lichen planus (LP) is a chronic inflammatory T-cell mediated disease affecting the skin, mucous membranes and skin appendages. Data on clinical phenotypes and systemic treatment of LP are limited. We analyzed a cohort of LP patients (equal or older than 18 years) regarding their clinical phenotypes (subtype, demographics, comorbidities) and treatment. Patients were selected from 2017 to 2023 who were seen in our outpatient clinic during a treatment period of at least 2 years. We identified 85 patients (62 females, 73% and 76 above 50 years; 89%) who met selection criteria. 33 had oral LP (39%), 23 had cutaneous LP (27%) and 2% had both or other manifestations (32%). Frequent comorbidities were hypertension (n = 40), hypothyroidism (n = 17), asthma (n = 11), diabetes (n = 10) and dyslipidemia (n = 8). 44% were taking a medication known to favor LP onset. 2/85 patients had a malignant transformation. 33 patients were treated topically and 50 required systemic therapy. Hydroxychloroquine (HCQ) (n = 18; 36%) and retinoids (n = 17; 34%) were the most commonly used systemic medications. Both were efficacious as determined by the investigator global response reaching rates 78% and 71%, respectively. The tolerability of retinoids was lower than HCQ (adverse event rate 29% versus 6%). Our results confirm previous data on clinical phenotypes and comorbidity patterns in LP patients. The treatment assessment suggests that HCQ may be an efficacious and safe first-line treatment for mucosal lichen planus. Further data from prospective controlled clinical trials are needed to prove the optimal treatment approach for different clinical phenotypes of LP.

## Introduction

Lichen planus (LP) is a chronic inflammatory T-cell mediated disease affecting the skin, mucous membranes and skin appendages [1].

**Fig. 1:**
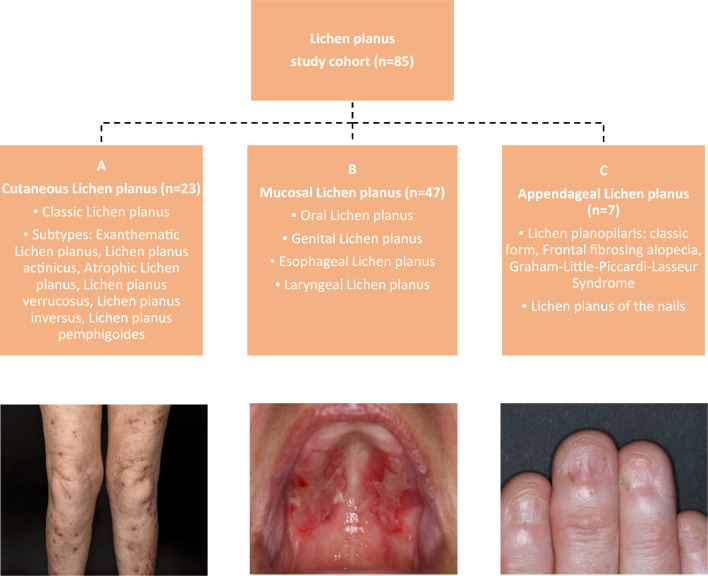
Clinical subtypes of lichen planus (LP); **A** polygonal dark-red lichenoid papules in a 75-year-old patient with cutaneous LP of the lower extremities; **B** painful and fibrotic erosions in a 85-year-old patient with erosive oral LP; **C** 73-year-old patient with nail LP presenting longitudinal ridging and pterygium formation

Clinically LP is differentiated into three main subtypes: mucosal lichen planus (MLP), cutaneous lichen planus (CLP) and appendageal lichen planus including lichen planopilaris (LPP), affecting the scalp, and nail lichen planus (Fig. 1). In most cases, patients suffer from one subtype, but multiple subtypes can coexist in up to 10% of affected patients [2]. Each subtype has heterogeneous manifestations. For instance, CLP classically presents dark red brownish polygonal papules. In other cases, the disease can occur with a wart-like elevated (LP verrucosus), atrophic (LP atrophicus), linear (LP striatus), exanthematic (LP exanthematicus) or bullous (LP pemphigoides) aspect. MLP can affect the oral mucosa (oral LP, OLP), the genital mucosa, or both [1]. In OLP, while some patients only have papules and whitish lace-like lesions (Wickham striae), some others have painful erosions that might also affect the esophageal and/or laryngeal epithelia and require strong systemic immunosuppressive treatment.

The prevalence of LP is estimated to be around 0.5—1.0% [3]. Most epidemiological data have been reported for OLP. Up to 0.89% of the general population worldwide have been suggested to suffer from this subtype with a predominance of women and adults over 40 years of age [4].

Immunologically, LP is characterized by a T-cell-dependent immune response resulting in apoptosis of basal keratinocytes. More recently, cytokines like Interferon (IFN)-γ, IFN-α and others such as IL-12 and IL-21 have been reported as dominant inflammatory mediators in this disease [5]. In line with these findings, drugs that specifically block IFN-γ/IFN-α signaling such as Janus kinase (JAK) inhibitors have been proven efficacious in LP [6–8].

While morphological characterization has been thoroughly performed, there is a lack and need to understand the priming factors leading to disease. Virus infection (hepatitis B and C, human papilloma virus) have been proposed as triggers of MLP and OLP [1]. Recently it was shown that skin antigens (e.g. bullous pemphigoid antigen BP180, desmoglein 3) are recognized by circulating T cells of patients with CLP and MLP [9]. Still, it is not known, which factors (genetic predisposition, priming factors) are responsible for the development of specific LP subtypes. The limited therapeutic options reflect this lack of knowledge. Topical treatment is mainly based on steroids, used as a cream for cutaneous lesions, mouthwash for OLP, or subcutaneous injections in verrucous LP. Alternatively, pimecrolimus or tacrolimus can be used to avoid steroid induced long-term side effects. Topical treatments can be combined with ultraviolet light therapy (UVB, Bath PUVA) in CLP. In certain patients, especially those affected by MLP, recalcitrant courses and non-responsiveness to topical treatment are observed. In these patients, steroids and steroid-sparing agents (i.e. azathioprine, methotrexate, dapsone) or vitamin A derivates (acitretin, isotretinoin) are used. Yet these compounds often show only partial results or lead to unbearable side effects [10, 11].

Hydroxychloroquine (HCQ) is a common and widely used immune-modulator and represents gold standard treatment for cutaneous and systemic lupus erythematodes. Immunologically, lupus erythematodes and LP share certain aspects, such as the presence of a marked IFN-γ/IFN-α signature. Hydroxychloroquine’s mechanism of action is not fully explained although recent data point towards a suppressive effect on IFN function [12, 13]. These aspects suggest that HCQ administration might be an effective treatment for LP.

There is still a lack of data regarding the clinical phenotypes and treatment regimens of LP. Herein, we present an analysis of 85 LP patients who were diagnosed and followed up in our outpatient clinic between 2017 and 2023. LP is a disease with different clinical phenotypes and comorbidity patterns, with a high prevalence in older adults and females. Among systemic treatment options, HCQ is hypothesized to be an effective and well-tolerated therapy, especially for mucosal LP.

## Methods

A retrospective observational cohort study was conducted. The medical records from patients (equal or older than 18 years) diagnosed with LP at our outpatient clinic between 2017 and 2023 were reviewed. Patients were identified by International Classification of Disease ICD-10 codes indicating a LP diagnosis (L43.0–L43.9). To analyze clinical response to treatments only patients visiting our clinic for more than 2 years were chosen according to the number of ICD-10 codes (7 or more ICD-10 codes for LP). The following inclusion criteria were applied: adults aged equal or older than 18 years, diagnosed with LP. Exclusion criteria included: children and young adults aged below 18 years, other dermatological diagnosis, insufficient follow-up (less than 7 ICD-10 codes for LP).

The primary objective of this study was to analyze the clinical phenotypes, demographic characteristics and comorbidity patterns in patients with LP. The secondary objective was to evaluate the efficacy and tolerability of systemic treatments in managing LP, particularly HCQ and retinoids. Demographic and clinical data including gender, age, type of LP, comorbidities, medication and treatment were therefore collected. Investigator Global Assessment (IGA) scores before and after systemic treatment with retinoids and HCQ were determined according to the documented clinical assessments by using an adjusted 5-point IGA grading system. Improvement was defined by a combination of objective clinical measures like reduction in lesion size or inflammation and patient-reported symptom relief. If a patient received multiple therapies, the last therapy was recorded. Descriptive statistics were used to summarize the data. Statistical analysis was performed using Microsoft Excel. Ethical approval for this study was obtained from the ethics committee of the Charité—Universitätsmedizin Berlin.

## Results

### Demographics and clinical subtypes

Of approximately 200.000 patients seen at our outpatient clinic from 2017 to 2023, 1204 (0.6%) were diagnosed with LP. A cohort of 91 patients was screened. After detailed review, 6 of the 91 patients did not meet our inclusion criteria. The final cohort consisted of 85 patients, which were further analyzed.

The majority of patients was equal or older than 50 years (n = 76; 89%). Women were predominantly affected (n = 62; 73% vs. n = 23; 27%). Histological examination was performed in n = 56 (66%), while in the other cases diagnosis was based on clinical appearance. One patient was hepatitis B positive.

MLP was present in 47 cases (55%), of which 33 cases had oral manifestation (39%) and 14 cases had both oral and genital manifestation (16%). Additionally, in MLP patients esophageal (n = 3) and laryngeal involvement (n = 1) was detected in 4 patients. 23 patients had CLP (27%), while 6 patients suffered from simultaneous cutaneous and oral involvement (7%). In CLP patients 11 were affected by exanthematic lichen planus (13%) and 4 with lichen planus verrucosus (5%), whereas the others had classic CLP. Appendageal LP was diagnosed in a total of 10 cases. 6 of these had LPP (7%) and 4 had nail lichen planus (5%).

### Comorbidities, medication and malignant transformation

The most frequently reported comorbidities were hypertension (n = 40; 47%), hypothyroidism (n = 17; 20%), asthma (n = 11; 13%), diabetes (n = 10; 12%) and dyslipidemia (n = 8; 9%). Depression was present in 6 patients. 44% of patients were taking medication known to cause LP, the most common being β-blockers (n = 22) and ACE inhibitors (n = 8). Grinspan’s syndrome (a syndrome characterized by the concomitant presence of essential hypertension, diabetes mellitus, and OLP) was present in four patients.

Malignant transformation of oral lichen planus to squamous cell carcinoma was detected in 2 out of 85 patients (2.4%). One patient developed a squamous cell carcinoma of the esophagus, the other patient developed an oral cancer of the tongue.

### Treatment

33 patients (39%) were treated with topical anti-inflammatory therapies, while 50 patients (59%) required a systemic therapy in combination with (n = 36; 42%) or without topical therapy (n = 14; 17%). 2 patients had no therapy.

**Fig. 2:**
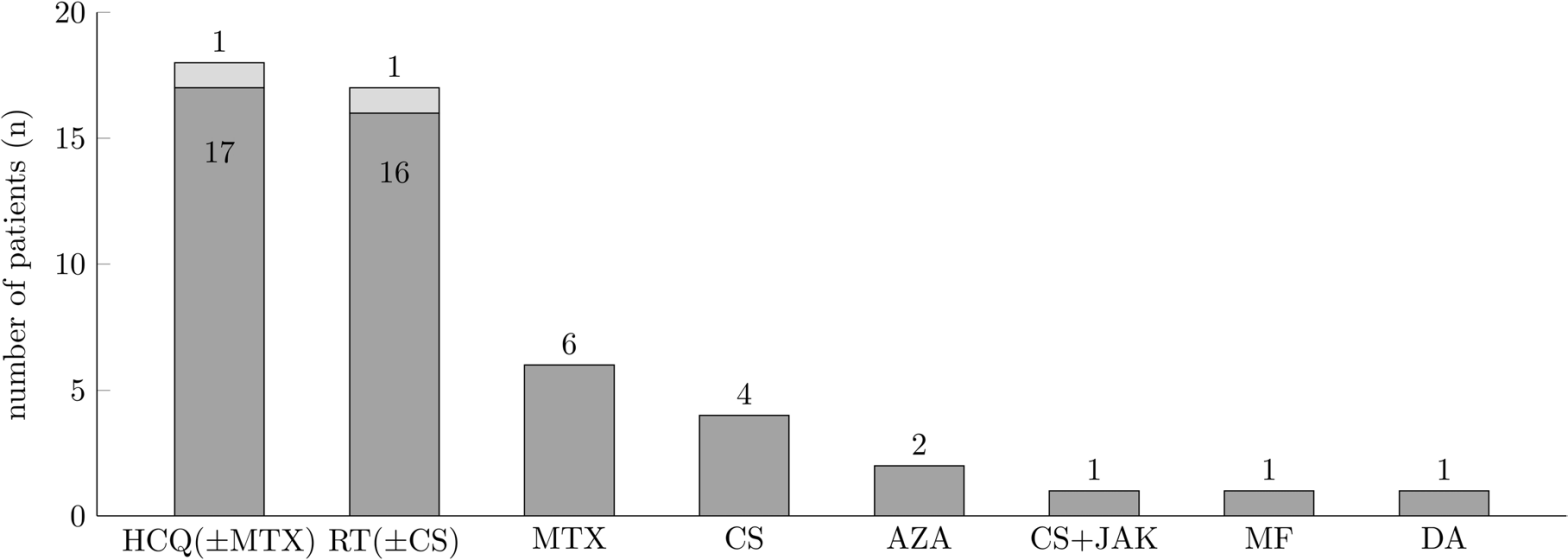
Spectrum and frequency of systemic therapies in patients with lichen planus (n = 50) (*HCQ* hydroxychloroquine, *MTX* methotrexate, *RT* retinoid, *CS* corticosteroids, *AZA* azathioprine, *JAK* Janus kinase inhibitor, *MF* mycophenolate mofetil, *DA* dapsone)

HCQ (n = 18; 36%) and retinoids (n = 17; 34%) were the most frequently administered systemic anti-inflammatory compounds (Fig. 2). Less frequently used were azathioprine, dapsone, mycophenolate mofetil monotherapy and combinations like systemic corticosteroids with JAK inhibitors or retinoids or HCQ with methotrexate.

**Fig. 3:**
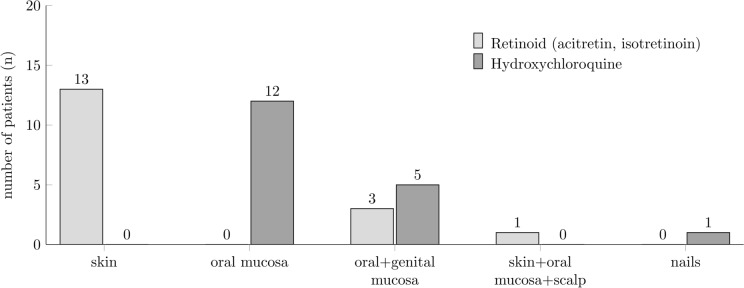
Clinical manifestations of patients with lichen planus treated with systemic retinoids or hydroxychloroquine

Subgroup analysis of patients treated with HCQ and retinoids showed that HCQ was primarily applied in patients with mucosal or nail involvement. Retinoids were used primarily in patients with CLP and in a few patients with MLP (Fig. 3).

### Efficacy and safety of HCQ and retinoids

**Fig. 4:**
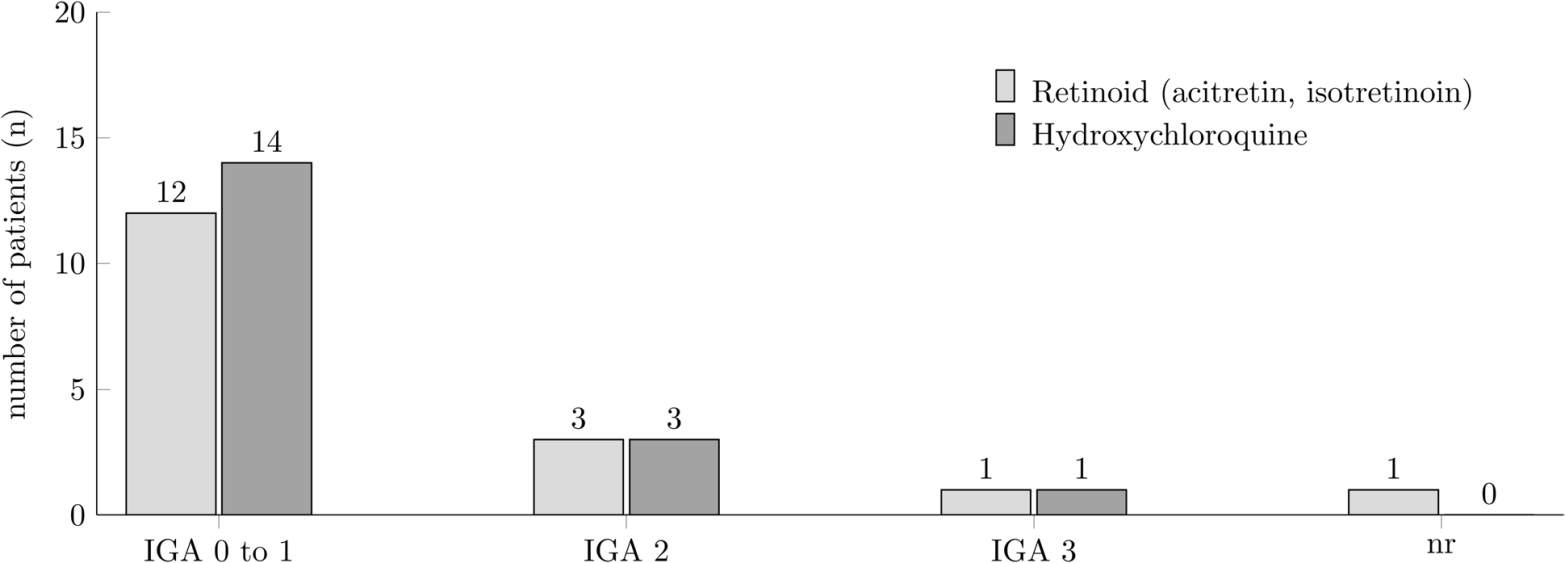
Investigator's Global Assessment Scale (IGA) after systemic treatment at least for 8 weeks with retinoids or hydroxychloroquine in patients with lichen planus

Next, we determined treatment responses using IGA scores (Fig. 4). 14/18 (78%) patients treated with HCQ responded with a decrease of IGA to 0 or 1. 4 patients had an improvement, but remained at higher IGA scores of 2 or 3. The dose of HCQ was 200 mg/day in 11 patients (61%) and 400 mg/day in 7 patients (39%).

Patients treated with retinoids had a slightly lower response rate of 12/17 (71%) patients reaching an IGA score of 0–1 (Fig. 4). Four patients had an IGA score of 2 or 3 while for one patient the clinical outcome was not reported. The dose of retinoids varied from 10 to 50 mg/day with 20 mg/day as the most frequent dose applied (n = 6; 35%).

### Adverse events

HCQ was overall very well tolerated leading only in one patient (6%) to an adverse event in the form of diarrhea. Meanwhile, 5/17 patients (29%) using retinoids suffered from adverse events like dryness of the skin and mouth but also hair loss and brittleness of nails.

## Discussion

In this retrospective analysis, we present a cohort of 85 patients with LP and report on their demographics, clinical characteristics and on their treatment approaches and outcomes. Significantly, we report that HCQ has a beneficial effect in LP, especially in OLP.

A recent review of LP patients from a German cohort selected from health insurance data also assessed demographics, comorbidities and treatments. In line with our results, these authors reported that women and patients between 60 and 79 years were frequently affected [2].

LP is a heterogeneous disease with a range of clinical subtypes, including cutaneous, mucosal, nail and scalp involvement. OLP was the most common type of LP in our cohort. Data from other studies show epidemiological discrepancy. Some studies reported CLP to be more frequent [14, 15], while one study also described OLP as more common [16]. The predominance of a given subtype may be influenced by the medical specialty seeing the patients, but also the severity of the disease. For instance, some forms of CLP might be very mild or asymptomatic and do not come in tertiary hospitals.

As many autoimmune diseases are more prevalent in women, estrogens may play a role in the pathogenesis of LP. From a clinical point of view, we propose that women with OLP should always receive a systematic exploration of genitals as this involvement may not be recognized [17].

Comorbidities like hypertension, hypothyroidism, asthma, diabetes mellitus and dyslipidemia were frequently observed in our cohort. An association between OLP and hypertension has been frequently reported in the literature [18]. Also, antihypertensive medication like ACE inhibitors and β-blockers have been suggested to promote the development of OLP. However, a coincidence of these factors cannot be ruled out as patients with hypertension are treated with antihypertensive drugs.

LP has also been previously linked to diabetes mellitus, dyslipidemia and metabolic syndrome. A cross-sectional study reported that these diseases were more common in LP patients [19]. Also, two recent systematic reviews suggested an association between diabetes/dyslipidemia and LP/OLP [20, 21]. The association of OLP with hepatitis C (HCV) is well accepted [22], although we identified only one patient with hepatitis B out of 56 screened patients. A positivity rate of up to 10% in LP patients has been reported and HCV screening is recommended in all patients [22].

According to the World Health Organization OLP is a potentially malignant disorder with an estimated malignant transformation ratio of 1.43% [23]. 2/85 patients (2,4%) of our cohort developed a squamous cell carcinoma which is concordant with the reported rates [16]. Screenings and biopsies of conspicuous lesions should be performed regularly in these patients.

The European S1 guideline summarizes first-, second- and third-line treatments for cutaneous, mucosal and appendageal LP [24]. The variety of treatments and the lack of FDA/EMA approved treatment implicates that LP is a challenging condition. Topical therapies are first line before an escalation to systemic treatments. Although application of topical corticosteroids is widely accepted, there is a lack of robust clinical evidence for their efficacy [25]. The majority of our patients 50/85 (59%) required a systemic treatment, while only 33 patients (39%) were sufficiently treated with topical therapies. Other reports indicate rates of 25–32% of LP patients requiring systemic therapy [14, 26]. A patient selection bias (university center) might be an explanation for this difference.

Topical, intralesional and systemic steroids, but also oral cyclosporine and acitretin are recommended as first-line therapies for CLP [24]. In line with these recommendations, retinoids (acitretin, isotretinoin) were used frequently in CLP (n = 13) and in few cases of MLP (n = 3).

Patients with MLP were mostly treated with HCQ (n = 17), which has been proposed in the S1 guideline as second-line therapy. Our findings suggest that HCQ had a slightly better clinical response rate and fewer side effects than retinoids. Other retrospective studies also confirmed the effectiveness of HCQ and recommended HCQ out of all steroid-sparing agents. One study recommends HCQ as a reserved treatment option due to side effects [14]. HCQ was well tolerated in our cohort, also in those patients receiving HCQ for long term periods. Both randomized controlled trials (RCT) and retrospective studies confirmed the efficacy and tolerability of HCQ in OLP [27, 28]. Significantly, HCQ has been proposed as an effective treatment for LPP as well [29]. Therefore, we propose HCQ to be considered as a first-line therapy in mucosal LP for patients, who do not sufficiently respond to topical anti-inflammatory treatments within 3 months.

The retrospective approach of our study has limitations, including a potential selection bias due to the university setting and missing data like timing of evaluation, duration of follow-up or any potential relapses post-treatment. Additionally, the IGA was based on clinical examination descriptions determined retrospectively. LPP has a separate ICD-10 code (L66.1) and was not considered in our study. Large, multi-center prospective studies are needed to determine a treatment algorithm for LP.

In conclusion, LP is a heterogeneous disease with a variable clinical presentation and a challenging treatment course. More studies are necessary to better understand the clinical efficacy of anti-inflammatory drugs in relation to the clinical subtype.

## Data Availability

The data that support the findings of this study are available from the corresponding author, KA, upon reasonable request.
